# Mechanisms of soil macrofauna community sustainability in temperate rice-growing systems

**DOI:** 10.1038/s41598-019-46733-4

**Published:** 2019-07-15

**Authors:** Daniil I. Korobushkin, Konstantin B. Gongalsky, Anastasia Yu. Gorbunova, Dmitry M. Palatov, Sergey V. Shekhovtsov, Andrei V. Tanasevitch, Julia S. Volkova, Sanal N. Chimidov, Elvira B. Dedova, Valery A. Ladatko, Tatiana V. Sunitskaya, Katharina John, Ruslan A. Saifutdinov, Andrey S. Zaitsev

**Affiliations:** 1 A.N. Severtsov Institute of Ecology and Evolution, Russian Academy of Sciences, Leninsky pr., 33, Moscow, 119071 Russia; 2 M.V. Lomonosov Moscow State University, Leninskie Gory, 1, Moscow, 119991 Russia; 3 Institute of Cytology and Genetics, Siberian Branch, Russian Academy of Sciences, Lavrientieva pr., 10, Novosibirsk, 630090 Russia; 4 Institute of Biological Problems of the North, Far Eastern Branch, Russian Academy of Sciences, Portovaya st., 18, Magadan, 685000 Russia; 5 Ulyanovsk State University, 100-letiya Lenina sq., 4, Ulyanovsk, 432700 Russia; 6 Federal State Unitarian Enterprise “Harada”, Lenina st., 1, Bolshoi Tsaryn, 359450 Russia; 7 Kalmykian Branch of Kostyakov All Russia Research Institute of Hydraulic Engineering and Land Reclamation, Gorodovikov sq., 1, Elista, 358011 Russia; 8 All-Russian Research Institute of Rice, Belozerny, 3, Krasnodar, 350921 Russia; 9 Primorsky Scientific Research Institute of Agriculture, Volozhenina st., 30, Timiryazevsky, Ussuriysk 692539 Russia; 10 Institute of Animal Ecology, Justus-Liebig-University, Heinrich-Buff-Ring, 26, Giessen, 35392 Germany; 11 Kazan Federal University, Kremlyovskaya str. 18, Kazan, 420008 Russia; 12 I.M. Sechenov First Moscow State Medical University, Institute of Molecular Medicine, Trubetskaya st., 8, Moscow, 119991 Russia

**Keywords:** Ecosystem ecology, Ecosystem ecology, Agroecology

## Abstract

Rice growing requires highly destructive and highly invasive field management negatively affecting soil biota and its functions. We aimed to compare taxonomic and functional trait compositions of soil macrofauna at different stages of rice cropping cycles in the three temperate rice-growing regions in Russia. Samples were collected in 2016 at four different biotopes in each region: flooded rice paddies; upland crops planted one year after flooded rice; rice paddy bunds; and relatively undisturbed seminatural control grasslands. Collected soil macrofauna were allocated to different traits according to their feeding preferences, vertical distribution, mobility and flood tolerance. The lowest macrofaunal abundance across all regions was observed in rice paddies. Cultivation of upland crops after paddy flooding consistently decreased the abundance of resident macrofauna, but not that of mobile soil macrofauna. In the upland crops, the abundance of belowground and mobile belowground macrofauna was significantly higher than that in control grasslands. The abundance of aboveground phytophages was significantly lower in the upland crops than in control sites. Flood-associated taxa showed low colonization ability after the paddies were drained. In contrast, representatives of other traits recorded in flooded fields increased their abundance at the next stage of crop rotation, demonstrating high resilience within an entire rice-growing system, including bunds. This finding indicates a high potential of seminatural grasslands and especially bunds as sources of rapid restoration of soil macrofauna functional diversity in rice-growing agroecosystems, thus maintaining the sustainability of soil food webs in the rice paddies.

## Introduction

Rice (*Oryza sativa* L.) is one of the most ancient cultivated plants and the staple food for the majority of the world’s population. Due to its high adaptation ability to a variety of environmental conditions, this cereal originating in the tropics is widespread and cultivated in temperate areas^1^. The cultivation of rice in these climatic conditions requires highly invasive and destructive land management measures and crop rotation cycles. However, recently efforts were made to find more ecologically-sound management practices involving biotic processes instead of intensive rice cultivation which generally results in the severe suppression of soil biota and soil fauna in particular. The former practices have the highest potential to contribute to increasing sustainability of rice growing in the temporarily flooded rice fields in the temperate regions^2,3^.

Soil fauna is one of the key ecological regulators in agroecosystems^4^. Being one of the most diverse groups in soil, macrofauna remain highly abundant, even after intensive agricultural practices and contains many important groups called ecosystem engineers^5^. Rice cultivation is particularly invasive with respect to soil ecosystems and adversely impacts soil properties^6,7^. Changes in physical and chemical conditions in paddy soils lead to substantial changes in the composition and trophic structure of soil macrofauna^8,9^. However, soil animal taxa could be pre-adapted to this agricultural practice, being resistant to flooding or soil mixing. At the same time, there are taxa that are strong invaders into disturbed ecosystems. Their ability to be resilient to long-lasting ecosystem transformation may play a key role in supporting the functioning of soil biota in rice-growing systems. There is growing concern that soil fauna may support soil ecosystem functioning^4^. It is important to maintain high level of functioning of indigenous soil fauna of agricultural ecosystems to meet the requirements of green agriculture and diminish the amounts of chemicals applied while cropping. Large soil invertebrates as integrators of soil processes are a key indicator of the ability of the entire soil food web to sustain various management measures.

Communities of soil macrofauna within rice paddy systems have been rarely studied around the world, and particularly rarely in non-tropical areas^10^. Overall, cropping systems may drastically change the relative abundance of soil macroinvertebrates that belong to different functional groups or have different functional traits^11,12^. Trait approach allows disentangling finer differences that are not revealed by assessing abundance and taxonomic diversity of soil fauna^13^. The latters are often used as indicators of various disturbances, while not always able to indicate the changes. At the same time, highly diverse systems as soil fauna is may not be compared by species number since there are taxa easily substituting each other after disturbances. In contrast, the trait approach is in fact a modeling of the number of ecological niches present in an ecosystem since all taxa of the same kind are bulked and analyzed as a whole. Trait approach is thus a finer tool to track not always detectable changes in a community. Thus, studying the functional and trait composition of belowground fauna seems important in order to reveal differences between various field treatments and crop rotation cycle stages. The trait approach is now considered to be among the most popular indicators of biodiversity and functional responses to both disturbances and natural gradients^14,15^.

In this study, we compared the taxonomic and functional trait composition of soil macrofauna at different stages of crop rotation within three key rice-growing regions in Russia, the Krasnodar Region, the Republic of Kalmykia in European Russia and the Primorsky Region in the Russian Far East to understand the potential of edaphic marcoinvertebrates persist in rice agrolandscapes. We studied the structure of macrofauna communities to assess the potential of surrounding seminatural grasslands and rice paddy bunds as sources for rapid recolonization for the rice paddies after their drainage. This preliminary knowledge will help us in the future to assess the functional potential of soil fauna to deliver necessary ecosystem services during rice cultivation.

For this we classified soil invertebrates by different flood tolerance traits: flood-associated (surviving flooding *in situ* and not increasing in abundance after field drainage), flood-tolerant quick recolonizers (encountered on both rice paddies and in upland crop fields with higher densities in the year following flooding), flood-intolerant quick recolonizers (found in the upland crop fields but not flooded rice), and flood-intolerant slow recolonizers (found neither in the upland crop fields nor in the flooded rice). We hypothesized that (i) taxonomically region-specific communities of soil macrofauna will demonstrate similar functional trait composition in the rice paddies and (ii) soil macrofauna present in the bunds that are able to quickly recolonize rice paddies after their drainage may play a major role in resurrection of soil macrofaunal communities, while species tolerant to flooding will be not abundant enough to maintain the diversity and functional structure of belowground communities. To our knowledge, this is the first attempt to disentangle soil macroinvertebrate communities in relation to their ability to withstand irrigation in the rice fields.

## Results

### Taxonomic composition of soil macrofauna

**Figure 1:**
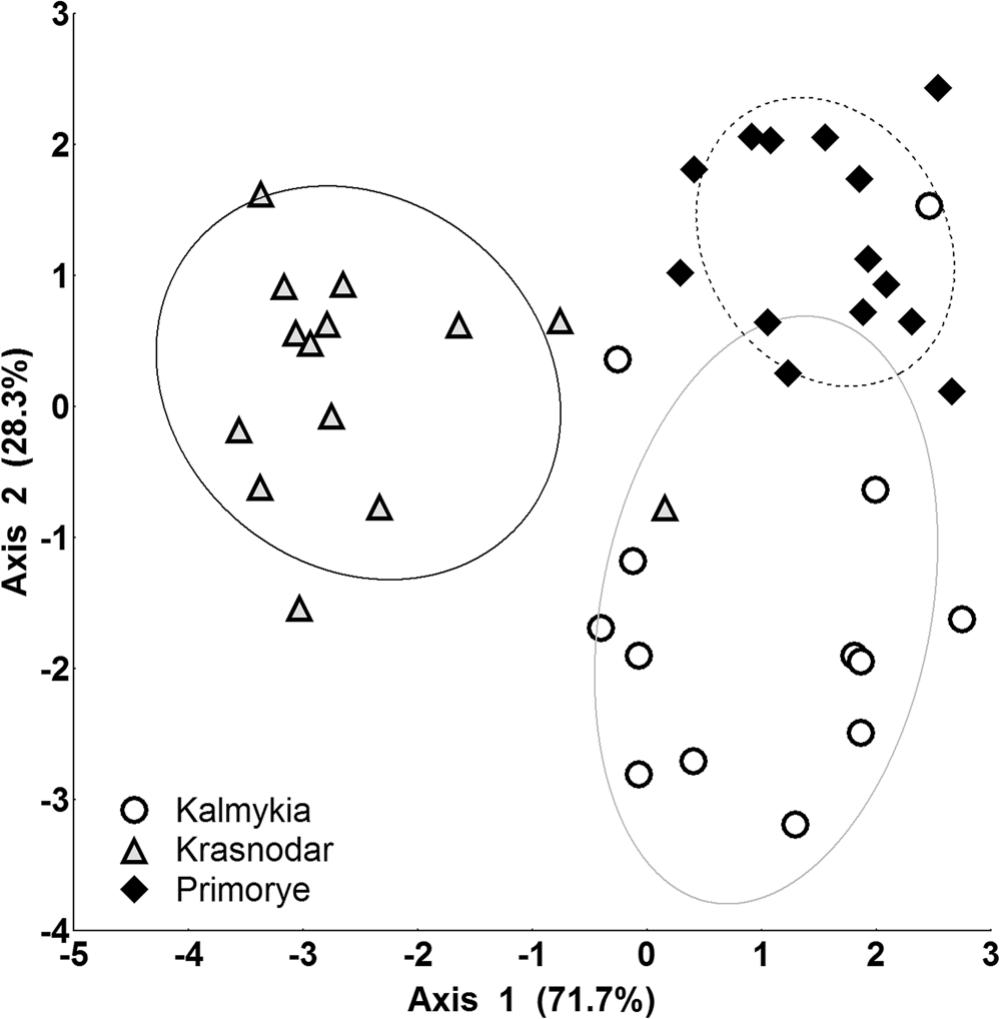
Multidimensional scaling with a subsequent canonical analysis of communities of soil macrofauna of three rice-growing regions. The dots indicate individual sites. The ellipses indicate the 95% confidence interval of the centroid position of a group.

The taxonomic composition of soil macrofauna communities in the three regions differed in the multidimensional space of the canonical analysis (Wilks’ Lambda: 0.09; F = 20.4, p < 0.0001) along two significant axes (Supplementary Table S1). Axis 1 (71.7% of the total explained variance) revealed the difference between Krasnodar and the other two regions (Fig. 1). Axis 2 (28.3% of explained variance) differentiated the faunistic composition of macrofauna mainly in Kalmykia and Primorye. Strong differences in the taxonomic composition between all three regions did not allow for the performance of an analysis of the distribution of taxonomic groups between individual sites (Wilks’ Lambda: 0.63, F = 1.4, p = 0.6).

### Total abundance and functional traits

**Figure 2:**
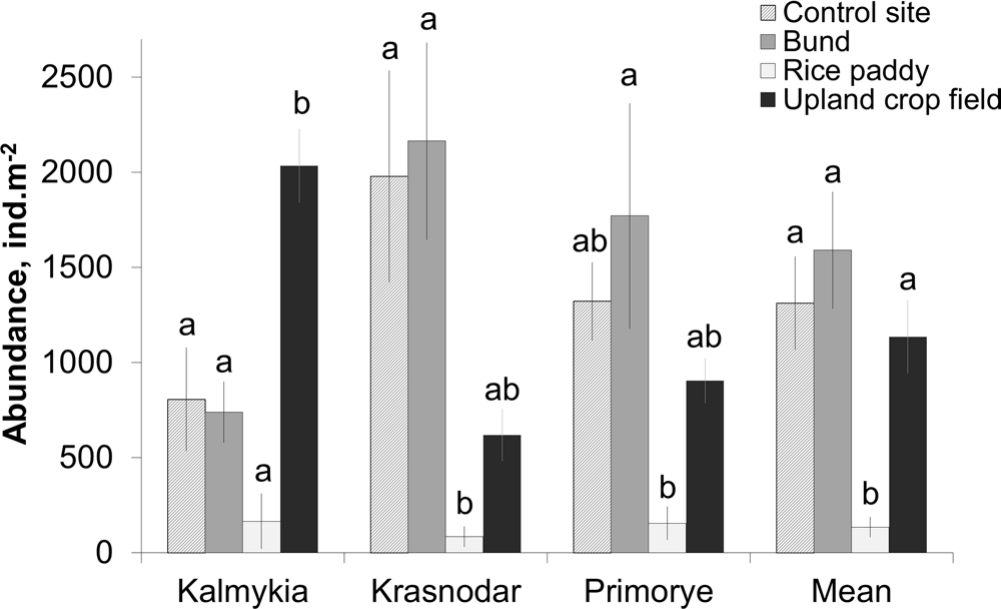
Total abundance of soil macrofauna (mean ± SE) within different biotope types across three study regions in Russia and average values for all sampling sites (Mean). Column marked with different letters indicate significant differences between the means according to the Tukey HSD post-hoc test (p < 0.05).

The total abundance of soil macrofauna differed significantly between biotope types (ANOVA: F = 10.0, p = 0.0001, Fig. 2). The lowest abundance across all regions was observed in the flooded rice paddies (Fig. 2). The interaction of the factors “Region” and “Biotope type” significantly affected the abundance of soil macrofauna (ANOVA: F = 4.0, p = 0.0046). However, in upland crop fields, the total abundance of macrofauna did not differ significantly from the control grasslands (Fig. 2). Unlike in the other two regions, in Kalmykia soil, macrofauna abundance in the upland crops was significantly higher than in the other biotope types (Fig. 2).

Flooding of rice paddies significantly reduced the abundance of the majority of macrofaunal traits and trait combinations in relation to control sites, regardless of region (Table 1). Exceptions were represented by the few groups with low abundance both in the control semi-natural grasslands and flooded rice paddies (e.g., resident aboveground phytophages and predators). The abundance of belowground and mobile saprophages in the flooded rice paddies did not significantly differ from that of the control grasslands (KW: H = 2.2, p = 0.13 and H = 2.4, p = 0.07 respectively). On the other hand, the flooding of rice paddies brought the abundance of belowground resident macroinvertebrates to zero. The abundance of soil macrofauna belonging to any trait combination did not significantly differ between bunds and control sites (Table 1).

**Table 1:** The abundance (ind.m^−2^) of macrofauna belonging to different traits and trait combinations within different rice growing system biotopes.

Trait/trait combinations	Rice paddy	Upland crop field	Bund	Control
Total abundance	135.6 ± 52.9^**^	1135.5 ± 193.2^ns^	1592.3 ± 306.7^ns^	1315.7 ± 243.8
Phytophages	18.9 ± 8.1^**^	293.4 ± 147.2^ns^	501.6 ± 161.7^ns^	507.4 ± 144.1
Predators	46 ± 12.6^**^	437.2 ± 108.3^ns^	492.7 ± 143.9^ns^	436.3 ± 106.7
Saprophages	69.6 ± 40^*^	286.2 ± 82.8^ns^	490 ± 190^ns^	316.7 ± 142.3
Unknown trophic position	1.2 ± 1.2	118.7 ± 46.1	107.9 ± 31.8	55.2 ± 16.8
Aboveground	53.1 ± 20.3^**^	579 ± 169.7^ns^	1095.2 ± 272.4^ns^	1038.2 ± 231.6
Belowground	82.6 ± 45.4^*^	454.1 ± 97.6^*^	391.8 ± 124.4^ns^	225.4 ± 35.3
Mobile	130.9 ± 52^**^	1003.7 ± 172.9^ns^	1059.8 ± 219.5^ns^	953.3 ± 186
Resident	4.7 ± 3.6^*^	29.5 ± 10.6^*^	427.2 ± 171.3^ns^	310.4 ± 166.4
Aboveground-Mobile	48.4 ± 17.1^**^	557.8 ± 171.4^ns^	707.7 ± 163.1^ns^	768.6 ± 172.6
Aboveground-Resident	4.7 ± 3.6^ns^	21.2 ± 10.5^ns^	386.6 ± 173.1^ns^	267.5 ± 166.4
Belowgound-Mobile	82.6 ± 45.4^*^	445.9 ± 96.2^*^	352.1 ± 122.4^ns^	184.7 ± 35.1
Belowgound-Resident	0^*^	8.3 ± 4.2^*^	39.7 ± 11.1^ns^	40.7 ± 9.9
Aboveground Phytophages	13 ± 7^**^	197.8 ± 140.2^*^	307 ± 87.8^ns^	428.9 ± 136.4
Belowground Phytophages	5.9 ± 5.9^*^	95.5 ± 40.3^ns^	194.6 ± 91.6^ns^	78.6 ± 20.3
Mobile Phytophages	17.7 ± 8.1^**^	290.5 ± 147.4^ns^	483 ± 160.8^ns^	498.9 ± 145.1
Resident Phytophages	1.2 ± 1.2^ns^	2.9 ± 1.5^ns^	18.6 ± 8^ns^	8.5 ± 5.4
Aboveground-Mobile Phytophages	11.8 ± 7^**^	194.9 ± 140.6^*^	288.4 ± 85.3^ns^	418.3 ± 136.8
Aboveground-Resident Phytophages	1.2 ± 1.2^ns^	2.9 ± 1.5^ns^	18.6 ± 8^ns^	8.5 ± 5.4
Belowground-Mobile Phytophages	5.9 ± 5.9^*^	95.5 ± 40.3^ns^	194.6 ± 91.6^ns^	78.6 ± 20.3
Aboveground Predators	33 ± 12.3^*^	308.8 ± 65.8^ns^	402.5 ± 149.8^ns^	337.6 ± 98.4
Belowground Predators	13 ± 5.5^**^	128.4 ± 75.7^ns^	90.2 ± 18.8^ns^	98.7 ± 21.8
Mobile Predators	46 ± 12.6^*^	429.5 ± 103.1^ns^	433.5 ± 146.2^ns^	367.3 ± 98.1
Resident Predators	0^*^	7.7 ± 6.7^*^	59.3 ± 14.9^ns^	69 ± 29.7
Aboveground-Mobile Predators	33 ± 12.3^*^	301.1 ± 65^ns^	371.5 ± 147.4^ns^	293 ± 87.7
Aboveground-Resident Predators	0^*^	7.7 ± 6.7^ns^	31 ± 12.8^ns^	44.6 ± 27.4
Belowground-Mobile Predators	13 ± 5.5^*^	128.4 ± 75.7^ns^	61.9 ± 18.2^ns^	74.3 ± 22.8
Belowground-Resident Predators	0^*^	0^*^	28.3 ± 11.6^ns^	24.4 ± 9.4
Aboveground Saprophages	7.1 ± 4.7^*^	72.4 ± 32.4^ns^	384.8 ± 184.4^ns^	270.7 ± 145.8
Belowground Saprophages	62.5 ± 41^ns^	213.8 ± 90^ns^	105.2 ± 44.9^ns^	46 ± 11.7
Mobile Saprophages	66.1 ± 40.5^ns^	267.3 ± 82.7^ns^	140.7 ± 58.1^ns^	83.9 ± 15.4
Resident Saprophages	3.5 ± 3.5^*^	18.9 ± 8.6^ns^	349.4 ± 163.5^ns^	232.9 ± 132.9
Aboveground-Mobile Saprophages	3.5 ± 1.8^*^	61.8 ± 31.9^ns^	46.9 ± 28.7^ns^	54.1 ± 18.4
Aboveground-Resident Saprophages	3.5 ± 3.5^ns^	10.6 ± 8.6^ns^	337.9 ± 160.3^ns^	216.6 ± 134.4
Belowground-Mobile Saprophages	62.5 ± 41^ns^	205.6 ± 87.5^ns^	93.8 ± 45.2^ns^	29.7 ± 6.3
Belowground-Resident Saprophages	0^ns^	8.3 ± 4.2^ns^	11.4 ± 5.3^ns^	16.3 ± 8.0

Statistical differences based on Kruskall-Wallis test of macrofauna abundance collected within following biotope pairs: control site vs. rice paddy; control site vs. upland crop field; control site vs. bund are shown in the table according to the following: *p < 0.05; **p < 0.001; ns – not significant. Comparisons were recognized significant at the p-level < 0.05. The “unknown trophic position” group was excluded from all of the statistical analyses of comparisons.

**Figure 3:**
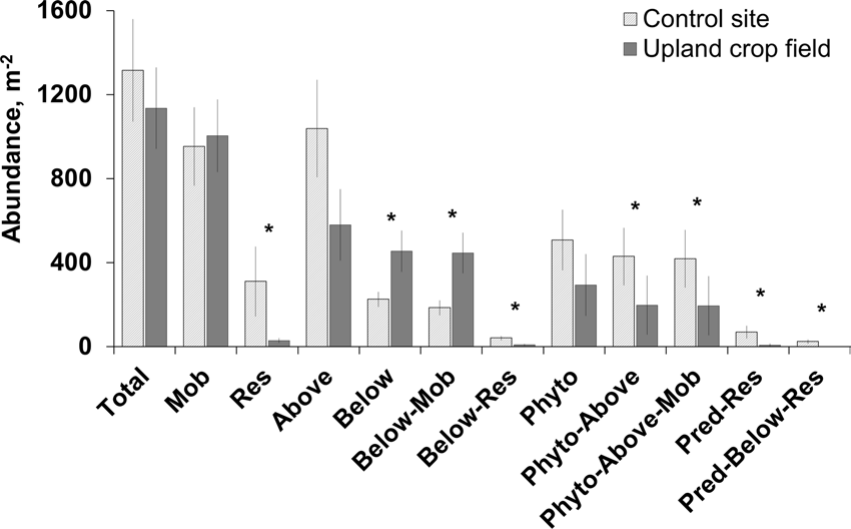
The abundance of macrofauna (mean ± SE) belonging to different traits within upland crop fields (solid gray bars) and control sites (hatched bars). Column pairs marked with an asterisk indicate significant differences between the means according to the KW H test (p < 0.05). Mob – mobile animals, Res – resident, Above – aboveground, Below – belowground, Phyto – phytophages, Pred – predators.

In the upland crop fields in the year after drainage, the abundance of resident animals was significantly lower (KW: H = 4.2, p = 0.04) compared with the control sites (Fig. 3). No significant differences were revealed for mobile invertebrates (KW: H = 0.12, p = 0.72). On the other hand, the abundance of belowground and mobile belowground macrofauna was weakly significantly higher within upland crop fields in comparison with control sites (KW: H = 3.8, p = 0.0486, Fig. 3). The abundance of aboveground phytophages was significantly (KW: H = 6.4, p = 0.01) lower within the upland crop fields compared to control sites (Fig. 3). The abundance of phytophages and especially of mobile and aboveground mobile phytophages was significantly lower within upland crop fields than in the control sites (Fig. 3).

### Macrofaunal tolerance to flooding during rice cultivation

**Figure 4:**
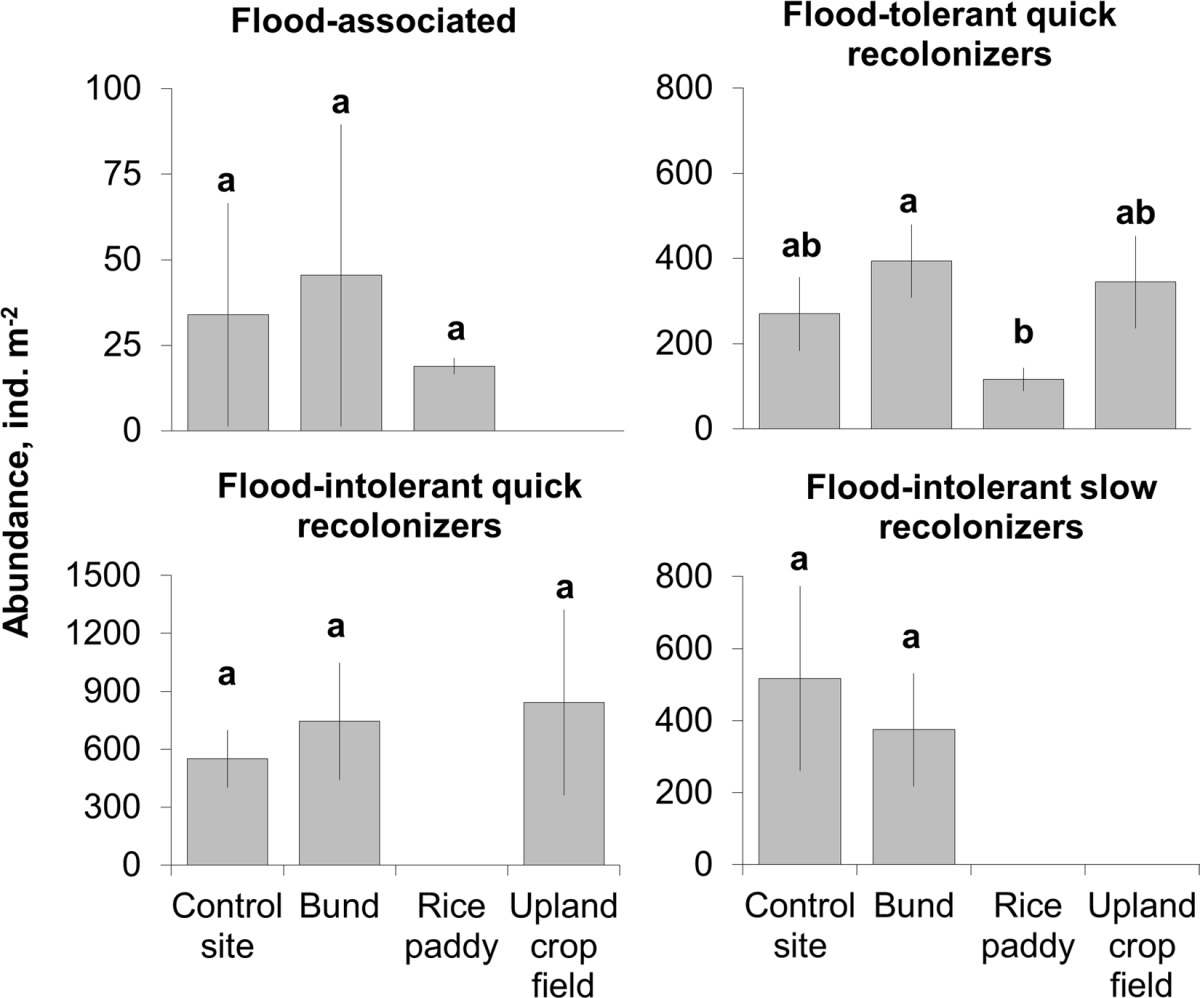
Abundance of soil macrofauna traits (mean ± SE) with respect to flood tolerance and colonization ability in flooded rice, upland crops, bunds and control habitats in rice-growing agroecosystems. Column marked with different letters indicate significant differences between the means according to the KW H test (p < 0.05).

The increase in soil macroinvertebrate abundance in the upland crops was mainly due to its quick recolonization by flood-intolerant groups. Flood-tolerant taxa contributed only to approximately one-third of the increase in total abundance of soil macrofauna in upland crops (Fig. 4). However, the abundance of the latter group in comparison with the flooded rice paddies significantly increased (KW: H = 3.8, p = 0.049) practically three-fold (from 116.8 ± 27 to 344.9 ± 107.8 individuals per m^−2^, further ind.m^−2^) (Fig. 4). The abundance of flood-intolerant slow colonizing taxa within control sites and bunds accounted for 550.9 ± 149.3 (or 40% of the total abundance) and 745.2 ± 301.8 ind.m^−2^ (48%), respectively, across all regions (Fig. 4). The abundance of all traits with respect to flood tolerance and recolonization ability did not significantly differ between bunds and control sites (KW: H > 0.5, p > 0.51). No regional effect was revealed for this parameter either in the bunds and control sites (KW: H = 7,1 p = 0,07; H = 7,2, p = 0,066 respectively); however, there was a tendency that flood-intolerant quick recolonizers dominated in the bunds, while in the control sites, their abundance was almost equal to that of flood-intolerant slow recolonizers.

## Discussion

We observed great differences in soil animal community composition across the three studied regions. In Kalmykia, indigenous soils bear a higher salt content, up to 0.4–0.6% (2.1–4.8 dS/m), and the highest aridity^16,17^, which probably leads to lower abundance and taxonomic richness of soil macrofauna compared to the soils of the other two regions. Overall, our observations on the diversity and faunistic composition of soil macroinvertebrates are in line with the earlier studies conducted in the areas we studied^10,18,19^.

It is generally known that flooding of rice paddies leads to a significant reduction of macrofaunal abundance (e.g., Lambeets *et al*.^20^; Coyle *et al*.^21^). This is consistent with our results indicating that during the flooded phase, the total macroinvertebrate abundance does not significantly differ from zero (Table 1, Supplementary Table S3). However, abundance fluctuations were highly trait specific. Despite the great faunistic dissimilarity revealed (Fig. 1), the functional response of soil communities to rice growing was highly consistent and independent of region. Of course, the revealed patterns cannot be automatically extrapolated to tropical rice agroecosystems or other temperate rice-growing areas due to the drastic differences in the practiced cropping cycles. In the tropics, for example, where the climatic conditions allow collecting a rice yield 2–3 times a year, soil fauna has practically no chance to establish a functional community^22^. The intervals between puddling may be as short as few weeks. At the same time, only a single rice yield could be harvested in the temperate climate conditions. There, the time period when the paddy is not flooded is much longer. For example, in the rice-growing systems we studied, flooding occurs for only three to four months every two or even three years. This makes rice production more “friendly” to soil biota, which is reciprocally supporting the potential of a larger volume of soil ecosystem functions provided by soil biota^6,23,24^.

Despite the fact that we had only a single snapshot of soil macrofauna composition at different habitats in the rice-growing landscapes. However, a space-for-time substitution method, which we effectively applied in our study^25^, clearly demonstrated that the most flood-tolerant groups were belowground mobile invertebrates especially saprophages, mainly represented by dipteran and coleopteran larvae or pupae (Supplementary Table 3). They may have even benefited from flooding as their natural enemies are adversely affected by submerged conditions^26^. Belowground resident animals (e.g., centipedes and earthworms), in contrast, do not tolerate flooding for long periods and thus were practically absent^27–31^. Mobile aboveground invertebrates, unlike the previous group, can rapidly immigrate to the flooded areas from neighboring ecosystems and then fold-back during the next flooded stage^20,32^. This might be the reason why the abundance of macrofauna and especially mobile and aboveground animals in the bunds was highest among other traits and was as high as in the control sites (Table 1). This further indicates that the bunds are a kind of refugia to survive inundation, as well as the further spreading and recolonization of animals back to the rice paddies.

Overall, animals possessing a high ability to survive flooding were mainly related to the water environment and only marginally connected to soil as their habitat (e.g., the Chironomidae, Ceratopogonidae larvae; Supplementary Table S3). Many of them occurred in our samples only accidentally. At the same time, these taxa showed low ability to survive and quickly restore abundance after the paddies were drained, what can be with some reservations interpreted as low resilience. Some other taxa occurred in the flooded fields but increased their abundance at the next stage of crop rotation, upland crops. Such a pattern suggests their resistance but also high resilience to disturbances associated with flooded rice growing. The joint contribution of these two groups to the overall functional resistance of soil macrofauna to flooding is quite high. In total, representatives of flood associated and flood-tolerant quick recolonizers accounted for 21 out of 34 possible functional trait combinations considered in our study. Other taxa, which quickly colonize drained fields, are not resistant but highly resilient to flooding by using bunds as refugia during flooding. Their high diversity and abundance proves the importance of semi-natural grasslands and bunds in maintaining the taxonomic and functional resilience of the overall macroinvertebrate community in the face of regular flooding disturbance.

In fact, rice-growing systems in temperate climate represent a unique experimental ground for testing the restoration of soil communities after drastic and regular disturbances of edaphic conditions. According to Pimm *et al*.^33^, two groups of organisms exist that can potentially support the recovery of disturbed communities. The first one includes those that survive disturbance *in situ* (flood-tolerant quick recolonizers in our case), and the second one combines those that recover in the longer run (flood-intolerant slow recolonizers). With our data, even applying a space-for-time substitution method with all its constrains^25^, we were able to disentangle such groups and quantify their relative importance in forming the community composition at different stages of crop rotation. Although our approach is still rather primitive and bears numerous limitations (e.g., based on the single observation time in the field, low taxonomic resolution of some groups, limited control over animal migration during observations, etc.), it sets the general framework for the assessment of the soil macrobiota potential to restore its structure and function after the field irrigation and drainage.

We conclude that in the conditions of bi- or triannual flooded rice cropping cycles in non-tropical agroecosystems, soil macrofauna communities have the potential to regularly restore their diversity and functional status. Bunds are important sources of potential rice paddy recolonization and refugia for soil biodiversity in the rice-growing areas, especially for aboveground mobile animals because they strongly contribute to the overall sustainability of soil macroinvertebrate assemblages. Belowground animals are the most tolerant to flooding in our study areas (Table 1) and ensure soil food-web survival *in situ*. The approach tested in our study could be further developed using long-term data on the occurrence of taxa in various biotope types. This will help to assess the functional response of soil biota to rice growing and estimate the role of soil macrofauna in supporting ecosystem services provided by rice-growing landscapes in the future.

## Material and Methods

### Sampling area and samples collection

Soil samples were collected once in each region in summer – early autumn of 2016 in each of three major rice-growing regions in Russia. Rice paddies of All-Russian Research Institute of Rice in Krasnodarsky Region (further referred to as “Krasnodar”) are located near the city of Krasnodar (45°04′N, 38°43′E; 17 m a.s.l.). This territory is characterized by a temperate moderately continental climate, with a mean annual temperature of 11.5 °C and mean annual precipitation of 675 mm^34^. The soils are Calcic Phaeozems^35^.

In the Republic of Kalmykia (“Kalmykia”), paddies of Federal State Unitarian Enterprise “Harada” and Kostyakov All Russia Research Institute of Hydraulic Engineering and Land Reclamation are located near the Bolshoi Tsaryn settlement, approximately 100 km south-east of the city of Volgograd (47°54′N, 45°21′E; 4 m a.s.l.). The locality is characterized by a semi-arid climate, with the mean annual temperature of 8.9 °C and mean annual precipitation of 313 mm^34^. Soils are Haplic Phaeozems^35^.

Paddies of Primorsky Scientific Research Institute of Agriculture in the Russian Far East, Primorsky Region, near the city of Ussuriysk (“Primorye”) (43°51′N, 131°56′E; 31 m a.s.l.) are characterized by a temperate monsoon climate, with a mean annual temperature of 3.3 °C and mean annual precipitation of 591 mm^34^. Soils are Umbric, Histic Fluvisols^35^.

In all three regions, rice is cultivated on artificially irrigated soil. The water is supplied from nearby large rivers, Kuban in Krasnodar, the Volga in Kalmykia, and Ussuri in Primorye. It is delivered through canals, and distributed across the paddies. The paddies are filled with water prior to rice seedling, and water table is maintained at the same level. The paddies are surrounded by bunds of up to 2 m high and 3–5 m width which are also used as tracks for agrimotors. Rice cropping in Russian system is based on planting seeds directly in the soil in contrast to plantings grown seedlings as done in the tropics. The practiced crop rotation cycle slightly differs between regions, but the typical scheme is as the follows: first year – one crop of flooded rice; second and third year – upland crop (alfalfa, soybean or wheat). Between May and August, paddies are flooded, with the water level set to a height of 20–25 cm, and drained just before harvesting, except for optional short-term drainage of paddy water. For draining, closing valves get unscrewed and water flows back into the channels surrounding the paddies. In addition, herbicides and pesticides are often used to protect crops from pests and diseases when the paddies are drained. However our study was conducted mainly within experimental agroecosystems of scientific agricultural institutes. Thus, mainly organic fields or fields with negligible amount of pesticides applied were selected.

Within each region, four biotope types were chosen and sampled (Supplementary Fig. S1): flooded rice paddy (hereinafter referred to as rice paddy), upland crop one year after rice (upland crop field), paddy bund (bund; artificial ramparts around rice paddies) and control site (control site) with semi-natural grassland vegetation (meadow or steppe depending on the region). In total, 14 sites in each region were selected. Vegetation descriptions, number of replicates and actual locations of each sampling site are provided in Supplementary Table S2. Climatic parameters and soil types are given according to Peel *et al*.^34^, the IUSS Working Group^35^ and the Russian Hydrometeorological Research Center public online database^36^.

To collect soil macrofauna, we obtained three intact soil cores at each site using a corer of 20 cm in diameter, down to the depth of 15 cm. In total, 126 soil cores were collected. Soil samples were drained immediately after collection, if needed, and delivered to the laboratory in cool boxes at a temperature of ca. +10 °C and set for extraction within 1–3 days after delivery.

### Samples processing

The extraction of macrofauna was performed using Tullgren extractors into a mixture of alcohol, water and ethylene glycol at a ratio of 80:15:5. A droplet of detergent was added to the resulting mixture to avoid surface tension. Extraction lasted for 7 days, the time sufficient for soil to reach air-dry conditions. Soil animals were sorted out from the samples under a binocular microscope and identified under a light microscope. To extract earthworms from the soil, the *in situ* formaldehyde method was applied^37^. Three 1-m^2^ areas were selected at each site, except for flooded paddies. All litter, if present, was thoroughly hand-sorted and removed, and all earthworms were collected from it. After that, 10 L of a 4% formaldehyde solution was sprayed over the area. All emerging earthworms were collected during a 20-min period after formaldehyde application and fixed in 95% alcohol.

### Functional trait analysis

All taxa were identified to a family, and some groups (Aranea, Oniscidea, Chilopoda and Diplopoda) were further identified to a species level. Soil macrofauna taxa were allocated to different traits according to their feeding preferences (saprophages, predators, and herbivores) following Gilyarov^18^, predominant vertical distribution (aboveground and belowground) and mobility (relatively mobile and predominantly resident) after Zaitsev *et al*.^38^. Some taxa were excluded from the trait analysis due to unknown trophic specialization and /or the impossibility of high-resolution taxonomic identification (e.g., coleopteran larvae and dipteran pupae) (Supplementary Table S3). The abundance of such taxa did not exceed 5% of soil macrofauna total abundance. We calculated the abundance of soil macroinvertebrates belonging to each of the possible trait combinations, including up to three traits describing each animal taxon. Overall, soil macrofauna was attributed to 34 out of 35 theoretically possible trait combinations. One combination (belowground resident phytophages) did not contain any taxa. The abundance of soil macrofauna was standardized to m^−2^ (individuals per m^−2^, further ind.m^−2^).

### Assessment of soil macrofauna tolerance to flooding and colonization ability after rice paddy drainage

Based on the occurrence and abundance of soil macroinvertebrates within different studied biotope types, we assessed the tolerance of different taxa to flooding and the ability to recolonize rice fields after their drainage. All soil macroinvertebrates were classified into four groups (Supplementary Table S3): (i) “*Flood-associated*” - taxa found in the flooded rice paddies (i.e., able to survive flooding *in situ*) and not significantly increasing their abundance in the upland crop fields; (ii) “*Flood-tolerant quick recolonizer*s” - taxa found in both rice paddies and upland crop fields the year following flooding with a significantly higher abundance in the upland crop fields; (iii) “*Flood-intolerant quick recolonizers*” - taxa that recovered in the upland crop fields (able to recolonize soil the next year after rice paddy drainage) but absent in the flooded rice; and (iv) “*Flood-intolerant slow recolonizers*”- taxa occurring neither in the upland crop fields nor in rice paddies but abundant in the bunds and control grasslands.

### Statistical analysis

Three soil samples within each site were considered pseudo-replicates and were only used to form a mean value per site. For each abundance value, the mean and standard error are provided based on results shown in Table S1. Statistical hypotheses were tested at the 0.05 significance p-level.

The taxonomic composition of soil invertebrate communities (at the family level) of the three regions was compared using the ordination method^39,40^. Samples with the total abundance of macrofauna equal to zero and with taxonomic richness lower than 3 taxa were excluded from the analysis. We calculated the matrix of all sites (n = 41) based on the relative abundance of macrofauna species and families. The similarity matrix was processed by multidimensional scaling (MDS). The optimum number of basic canonical axes was determined by the comparison of the actual and theoretical stress values. The obtained coordinates of points in the multidimensional space were used to assess the value and significance of differences between samples taken from different plots using a discriminant factor analysis (DFA). The significance of results was estimated by Wilks’ Lambda. When the DFA confirmed significant differences between the sites, the Spearman correlation between the ratio of the contribution to the total macrofauna abundance (%) of separate taxa in samples and the coordinates of these samples on the significant canonical axes were calculated.

Regional differences in the total abundance of macrofauna were tested using a Factorial ANOVA (n = 42) with main factors “Region” and “Biotope type”. The Factorial ANOVA was run on the untransformed data, because the Shapiro-Wilk’s test did not reject normality (p < 0.05) for these datasets. To test the significance of the effect of biotope types on trait combinations (rice paddy, upland crop field and bund), values were compared with control sites using a nonparametric Kruskal-Wallis H test (hereafter referred to as KW). The sample size of the compared pairs was as follows: rice field vs. control, n = 19; upland crop field vs. control, n = 22; and bund vs. control, n = 21. All statistical analyses were performed using the STATISTICA 13.0 software (TIBCO Software Inc., Tulsa, USA), and p < 0.05 indicated statistical significance.

## Supplementary information

Supplementary materials

Table S3
