# Supplementary material for: Mechanisms of soil macrofauna community sustainability in temperate rice-growing systems
Source: Sci Rep. 2019 Jul 15;9:10197. doi: 10.1038/s41598-019-46733-4 (PMC6629642; doi:10.1038/s41598-019-46733-4)
Supplement: Supplementary file 1 — Supplementary materials [file 41598_2019_46733_MOESM1_ESM.pdf]

Mechanisms of soil macrofauna community sustainability in temperate rice-growing systems

Daniil I. Korobushkin[1]*, Konstantin B. Gongalsky[1,2], Anastasia Yu. Gorbunova[1,2], Dmitry M. Palatov[1,2], Sergey V. Shekhovtsov[3,4], Andrei V. Tanasevitch[1], Julia S. Volkova[5], Sanal N. Chimidov[6], Elvira B. Dedova[7], Valery A. Ladatko[8], Tatiana V. Sunitskaya[9] Katharina John[10], Ruslan A. Saifutdinov[1,11] and Andrey S. Zaitsev[1,10,12]

[1] *A.N. Severtsov Institute of Ecology and Evolution, Russian Academy of Sciences, Leninsky pr., 33, Moscow, 119071, Russia*
[2] *M.V. Lomonosov Moscow State University, Leninskie Gory, 1, Moscow, 119991, Russia*
[3] *Institute of Cytology and Genetics, Siberian Branch, Russian Academy of Sciences, Lavrientieva pr., 10, Novosibirsk, 630090, Russia*
[4] *Institute of Biological Problems of the North, Far Eastern Branch, Russian Academy of Sciences, Portovaya st., 18, Magadan 685000, Russia*
[5] *Ulyanovsk State University, 100-letiya Lenina sq., 4, Ulyanovsk, 432700, Russia*
[6] *Federal State Unitarian Enterprise "Harada", Lenina st., 1, Bolshoi Tsaryn, 359450, Russia*
[7] *Kalmykian Branch of Kostyakov All Russia Research Institute of Hydraulic Engineering and Land Reclamation, Gorodovikov sq., 1, Elista, 358011, Russia*
[8] *All-Russian Research Institute of Rice, Belozerny, 3, Krasnodar, 350921, Russia*
[9] *Primorsky Scientific Research Institute of Agriculture, Volozhenina st., 30, Timiryazevsky, Ussuriysk, 692539, Russia*
[10] *Institute of Animal Ecology, Justus-Liebig-University, Heinrich-Buff-Ring, 26, Giessen, 35392, Germany*
[11] *Kazan Federal University, Kremlyovskaya str. 18, Kazan, 420008, Russia*
[12] *I.M. Sechenov First Moscow State Medical University, Institute of Molecular Medicine, Trubetskaya st., 8, Moscow, 119991, Russia*

* Dkorobushkin@yandex.ru

Supplementary materials

Figure S1. A panoramic view of sampling sites in Krasnodar region (Photo by: D.I. Korobushkin).

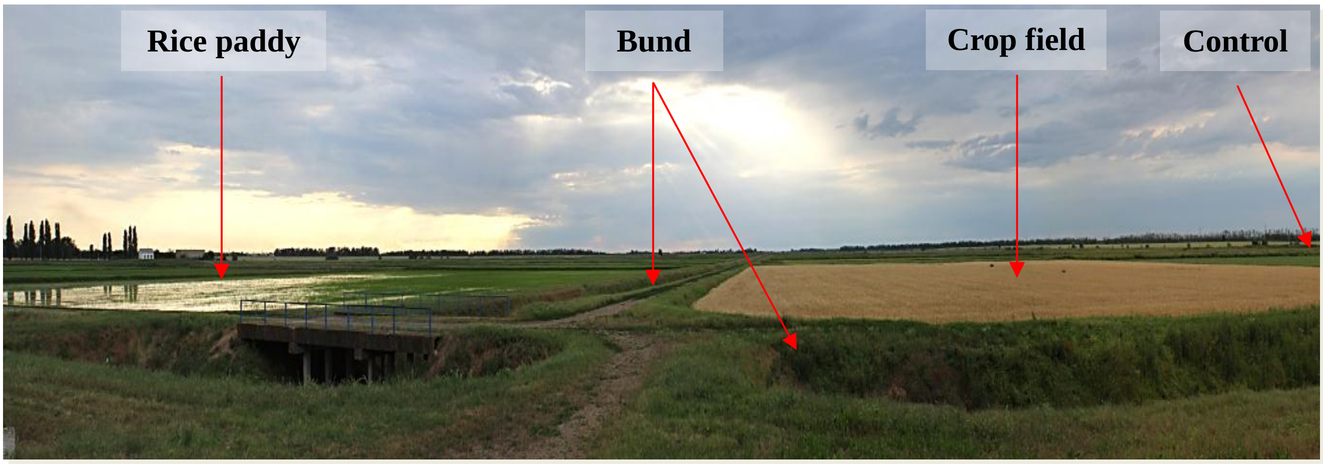

Table S1. Spearman correlation (r-values) between relative abundance of taxa and discriminant axes 1 and 2. The significance of correlations is shown in table according to the following: * - p<0.05; ** - p<0.001; *** - p<0.0001; ns – not significant. Acronyms: L – larvae; fam. – unknown family, number after fam. indicates different families within one order.

Taxa	Axis 1	Axis 2
Agriolimacidae	-0,74^{ns}	0,0^{ns}
Alleculidae	-0,7*	0,0^{ns}
Anisopodidae	-0,66^{ns}	0,0^{ns}
Aphidoidea	-0,52^{ns}	0,0^{ns}
Asilidae	-0,51^{ns}	0,0^{ns}
Cantharidae	-0,49*	0,0^{ns}
Carabidae	-0,43^{ns}	0,0^{ns}
Carabidae L	-0,43^{ns}	0,0^{ns}
Cecidomyiidae	-0,4^{ns}	0,01*
Cerambycidae	-0,39*	0,01^{ns}
Ceratopogonidae	-0,38^{ns}	0,01*
Chironomidae	-0,38**	0,02^{ns}
Chordeumatidae	-0,37*	0,02^{ns}
Chrysomelidae	-0,36^{ns}	0,02^{ns}
Cicadellidae	-0,33^{ns}	0,03**
Coccidae	-0,33^{ns}	0,04^{ns}
Coleoptera L fam. 01	-0,29**	0,06^{ns}
Coleoptera L fam. 03	-0,28*	0,08^{ns}
Coleoptera L fam. 05	-0,27^{ns}	0,09*
Coleoptera L fam. 06	-0,25^{ns}	0,11***
Coleoptera L fam. 07	-0,23^{ns}	0,15*
Coleoptera L fam. 08	-0,22^{ns}	0,16*
Coleoptera L fam. 09	-0,17*	0,29*
Coleoptera L fam. 11	-0,15^{ns}	0,33^{ns}
Coleoptera fam. 01	-0,14^{ns}	0,4*
Coleoptera fam. 07	-0,1^{ns}	0,54**
Curculionidae	-0,09*	0,56***
Curculionidae L	-0,08^{ns}	0,61*
Cylisticidae	-0,07^{ns}	0,66^{ns}
Diaspididae	-0,03*	0,84^{ns}
Diptera L fam. 01	0,06^{ns}	0,71^{ns}
Dolichopodidae	-0,03^{ns}	0,85**
Elateridae	-0,03^{ns}	0,87^{ns}
Elateridae L	-0,02^{ns}	0,91*
Geophilidae	0,01^{ns}	0,94*
Gnaphosidae	0,02^{ns}	0,89^{ns}
Hahniidae	0,03^{ns}	0,83^{ns}
Julidae	0,05***	0,75^{ns}
Lagriidae L	0,23^{ns}	0,14^{ns}
Lauxaniidae	0,07^{ns}	0,68^{ns}
Linyphiidae	0,07*	0,68*

Lithobiidae	0,07*	0,65^{ns}
Lophoproctidae	0,08***	0,61^{ns}
Lumbricidae	0,08^{ns}	0,61*
Lycosidae	0,08^{ns}	0,61^{ns}
Lygaeidae	0,05***	0,77^{ns}
Muscidae	0,11^{ns}	0,51^{ns}
Mycetophilidae	0,11^{ns}	0,48^{ns}
Noctuidae	0,12^{ns}	0,44^{ns}
Oedemeridae L	0,12^{ns}	0,44*
Paradoxosomatidae	0,14^{ns}	0,4^{ns}
Phlaeothripidae	0,14^{ns}	0,38^{ns}
Phrurolithidae	0,14^{ns}	0,38^{ns}
Polydesmidae	0,14*	0,38^{ns}
Psychodidae	0,15^{ns}	0,36^{ns}
Ptiliidae	0,16^{ns}	0,33^{ns}
Rhagionidae	0,18**	0,27^{ns}
Scarabaeidae	0,18^{ns}	0,27^{ns}
Scarabaeidae L	0,18^{ns}	0,26^{ns}
Scatopsidae	0,23^{ns}	0,14^{ns}
Staphylinidae	0,27^{ns}	0,09*
Staphylinidae L	0,31**	0,05^{ns}
Stratyomyiidae	0,32^{ns}	0,04^{ns}
Tabanidae	0,32*	0,04*
Tenebrionidae L	0,44^{ns}	0**
Thripidae	0,49*	0,0^{ns}
Trachelipodidae	0,53*	0,0^{ns}
Valloniidae	0,55*	0,0^{ns}

Table S2. Detailed description of the sampling sites in three rice growing regions of Russia.

#	Biotope type	Replicate	Nearest locality	Coordinates N	E	m a.s.l.	Vegetation
				**Krasnodar Krai (Krasnodar)**			
1	Bund	1	Risoopyny	45°13’40”	38°18’51”	6	Reed-cereal
2	Bund	2	Mogukorovskiy	45°08’06”	38°11’49”	2	Grassland
3	Bund	3	Mogukorovskiy	45°08’06”	38°11’49”	2	Reed
4	Bund	4	Kalininskaya	45°30’22”	38°36’02”	6	Grassland with reed
5	Bund	5	Kalininskaya	45°30’22”	38°36’02”	6	Grassland
6	Control site	1	Risoopyny	45°14’41”	38°17’25”	8	Grassland
7	Control site	2	Troitskoe	45°06’55”	38°06’29”	1	Wet Grassland with sedge
8	Control site	3	Kalininskaya	45°26’42”	38°36’52”	3	Grassland
9	Upland crop	1	Risoopyny	45°13’40”	38°18’50”	6	Soya
10	Upland crop	2	Mogukorovskiy	45°08’06”	38°11”50”	2	Soya
11	Upland crop	3	Kalininskaya	45°30’22”	38°36’02”	5	Soya
12	Rice paddy	1	Risoopyny	45°13’40”	38°18’51”	6	Rice
13	Rice paddy	2	Mogukorovskiy	45°08’06”	38°11”50”	2	Rice
14	Rice paddy	3	Kalininskaya	45°30’22”	38°36’02”	5	Rice
				**Republic of Kalmykia (Kalmykia)**			
15	Bund	1	Bolshoi Tsaryn	47°54’43”	45°22’31”	7	Reed
16	Bund	2	Bolshoi Tsaryn	47°54’43”	45°22’31”	7	Reed
17	Bund	3	Bolshoi Tsaryn	47°54’43”	45°22’31”	7	Reed with festuca
18	Bund	4	Bolshoi Tsaryn	47°47’57”	45°23’35”	6	Reed
19	Control site	1	Bolshoi Tsaryn	47°54’19”	45°20’46”	4	Steppe with sagebrush, cereals and saltwort
20	Control site	2	Bolshoi Tsaryn	47°54’19”	45°20’46”	4	Steppe with sagebrush, cereals and saltwort
21	Control site	3	Bolshoi Tsaryn	47°46’44”	45°22’52”	7	Steppe with sagebrush, cereals and saltwort
22	Control site	4	Bolshoi Tsaryn	47°47’57”	45°23’35”	6	Grassland with sagebrush
23	Upland crop	1	Bolshoi Tsaryn	47°54’43”	45°22’31”	7	Fall wheat
24	Upland crop	2	Bolshoi Tsaryn	47°54’43”	45°22’31”	7	Alfalfa
25	Upland crop	3	Bolshoi Tsaryn	47°54’43”	45°22’31”	7	Alfalfa with reed and thistle
26	Rice paddy	1	Bolshoi Tsaryn	47°54’43”	45°22’31”	7	Rice
27	Rice paddy	2	Bolshoi Tsaryn	47°54’43”	45°22’31”	7	Rice
28	Rice paddy	3	Bolshoi Tsaryn	47°47’57”	45°23’35”	6	Rice

Primorsky Krai (Primorye)

29	Bund	1	Timiryazevsky	43°51’34”	131°53’54”	29	Grassland
30	Bund	2	Timiryazevsky	43°51’34”	131°53’54”	29	Grassland
31	Bund	3	Lugovoy	44°32”59”	132°07’58”	85	Grassland with reed
32	Control site	1	Timiryazevsky	43°51’34”	131°53’54”	29	Grassland
33	Control site	2	Timiryazevsky	43°52”14”	131°57’24”	29	Grassland
34	Control site	3	Lugovoy	44°32’59”	131°07’58”	85	Grassland
35	Upland crop	1	Timiryazevsky	43°51’34”	131°53’54”	29	Wheat
36	Upland crop	2	Timiryazevsky	43°51’34”	131°53’54”	29	Soya
37	Upland crop	3	Timiryazevsky	43°51’34”	131°53’54”	29	Wheat
38	Upland crop	4	Timiryazevsky	43°51’34”	131°53’54”	29	Soya
39	Upland crop	5	Lugovoy	44°32’59”	131°07’58”	85	Soya
40	Rice paddy	1	Timiryazevsky	43°51’34”	131°53’54”	29	Rice
41	Rice paddy	2	Timiryazevsky	43°51’34”	131°53’54”	29	Rice
42	Rice paddy	3	Lugovoy	44°32’59”	131°07’58”	85	Rice
